# Comparative efficacy of psychological, psychosocial, and community-level interventions for antenatal depression: systematic review and network meta-analysis

**DOI:** 10.1192/j.eurpsy.2026.11673

**Published:** 2026-07-31

**Authors:** A. Melillo, E. Caporusso, B. Della Rocca, C. Toni, S. Calamaro, M. Luciano, A. Fiorillo

**Affiliations:** University of Campania “L. Vanvitelli”, Napoli, Italy

## Abstract

**Introduction:**

Peripartum depression affects 10-20% of women and has significant consequences for both maternal and child health and. During pregnancy, pharmacological treatments pose concerns due to foetal exposure and limited acceptability, highlighting the need for safe and effective alternatives. While evidence supports psychological and psychosocial approaches, their comparative efficacy and acceptability remain unclear. Current evidence is fragmented by intervention type, as comparisons across different levels of care (psychological, psychosocial, and community-based) are lacking (Cuijpers et al. Psychol Med. 2023;53(6):2596-60; Yin et al. J Aff Disorders 2020;271:310-27). Most available data pertain to peripartum depression, leaving antenatal-specific evidence underdeveloped. Finally, studies often relied on self-reporting screening tools, which produce high false-positive rates (Levis et al. BMJ 2020;371:m4022), confounding estimates of the intervention’s efficacy.

**Objectives:**

To systematically compare the efficacy and acceptability of psychological, psychosocial, and community-based interventions for the treatment of antenatal depression.

**Methods:**

We searched PubMed, Scopus, WoS, and PsycINFO for RCTs enrolling pregnant women with DSM/ICD-diagnosed depression and comparing non-pharmacological intervention against either control (treatment-as-usual or waitlist) or other active interventions. Primary outcomes were depressive symptom reduction and remission and acceptability. Random-effects pairwise and network meta-analyses will be performed. Risk of bias will be assessed via RoB-2 tool, and the certainty of evidence via GRADE.

**Results:**

Database searches produced 1,178 records. A total of 45 articles were assessed for eligibility. 15 RCTs met inclusion criteria (excluded articles, n = 30: wrong study design, n = 5; wrong diagnosis, n = 20; lack of control group, n = 5). A total of 12 intervention types were included (psychological, n=6; psychosocial, n=4; community-based, n=2). Most studies were conducted in high-income countries. Sample sizes (mean = 100; range 34–903 participants) and methodologies varied. Data extraction and analysis are still ongoing, and results will be presented at the Congress.

**Conclusions:**

To the best of our knowledge, this is the first NMA to compare the efficacy of psychological, psychosocial, and community-level interventions for antenatal depression, aiming to identify comparative hierarchies of intervention effectiveness and acceptability. We expect our findings to inform on non-pharmacological strategies for antenatal depression and promote the integration of community-based and psychosocial programmes with psychological therapies in perinatal care.

**Disclosure of Interest:**

None Declared

